# Cage‐Type Porous Organic Salts as Ship‐in‐a‐Bottle Nanoreactors for Light‐Transparent and Reusable Molecular Photocatalysts

**DOI:** 10.1002/anie.6654151

**Published:** 2026-06-10

**Authors:** Kazuki Shiga, Ryosuke Nishikubo, Masafumi Minoshima, Akinori Saeki, Kazuya Kikuchi, Norimitsu Tohnai

**Affiliations:** ^1^ Department of Applied Chemistry Graduate School of Engineering The University of Osaka Suita Osaka Japan

**Keywords:** homogeneous catalysis, light‐transparent nanoreactors, molecular photocatalysts, photocatalysis, porous organic salts

## Abstract

Heterogenizing molecular photocatalysts while preserving their intact, pre‐designed structures—without redesigning the host—remains a fundamental challenge. Immobilization within porous materials offers control over reaction environments and improved recyclability; however, most host systems require catalyst‐specific structural redesign, limiting generality. We introduce a cage‐type porous organic salt (POS) as a modular host platform for homogeneous, molecularly defined photocatalysts (organic molecules and metal coordination complexes) based on a ship‐in‐a‐bottle strategy. A sodalite‐type POS constructed from adamantane‐based sulfonic acid and tri(ethynylphenyl)methylamine provides well‐defined, light‐transparent, cage‐like internal cavities. Single‐crystal X‐ray diffraction reveals that the POS accommodates diverse photocatalysts, including anthraquinone, phenanthrenequinone, and phthalocyanine, through a unified recrystallization protocol. The framework exhibits negligible absorption in the visible region, enabling selective excitation of photocatalysts without competitive light absorption. Post‐synthetic thiol–yne crosslinking of ethynyl groups on the pore surface reinforces the framework and renders it insoluble while preserving cage‐like cavities. For example, a phenanthrenequinone‐encapsulated POS functions as a reusable heterogeneous photocatalyst for visible‐light‐driven hydrogen peroxide production from isopropanol and oxygen, achieving rates of up to 4.2 mmol g^−1^ h^−1^ without catalyst leaching. Overall, cage‐type POSs serve as light‐transparent nanoreactors for intact molecular photocatalysts, providing a modular, expandable platform for photocatalyst immobilization and post‐synthetic functionalization.

## Graphical abstract

Light‐transparent, cage‐type porous organic salts (POSs) function as nanoreactors that heterogenize molecularly defined photocatalysts through size exclusion. Photocatalysts are encapsulated intact during crystallization, providing confined yet accessible reaction fields under visible light without catalyst modification. This proof‐of‐concept study establishes POS nanoreactors as a general platform for heterogeneous photocatalysis.

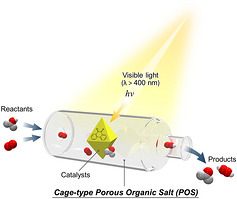

## 1 Introduction

Photocatalytic reactions play a central role in various fields, such as environmental remediation, chemical synthesis, and energy conversion; hence, improving their efficiency and stability remains an important challenge [1, 2, 3, 4]. Homogeneous, molecularly defined photocatalysts often exhibit high reactivity owing to precise molecular‐level design. However, their practical application is hindered by difficulties in catalyst recovery and reuse, as well as susceptibility to deactivation resulting from concentration‐dependent dimerization [5] and self‐quenching [1, 6, 7, 8]. These limitations highlight the need for innovative solid‐state reaction fields that enable the heterogenization of photocatalysts while maintaining their intrinsic functions through appropriate spatial isolation.

To date, diverse materials, including inorganic oxides such as SiO_2_, carbon‐based materials, and polymeric materials, have been used as support for photocatalysts [9, 10, 11]. However, in these systems, catalyst molecules are typically immobilized through surface adsorption or covalent bonding, making it difficult to uniformly control their spatial arrangement and local environment at the molecular level [12, 13, 14]. Consequently, complete suppression of catalyst aggregation and deactivation remains challenging.

Crystalline porous materials, especially metal–organic frameworks [4, 15, 16, 17] and covalent organic frameworks [18, 19, 20, 21], enable catalyst immobilization within precisely defined pore architectures while maintaining high surface areas. However, because catalytic moieties are typically incorporated into the framework backbone, the host structure must be redesigned for each photocatalyst [22]. Moreover, many photocatalysts cannot be introduced into these frameworks in their intact, pre‐designed molecular forms and instead require ligand redesign [23], anchoring functionalization [24, 25], or reconstruction of framework metal nodes [26]. Consequently, the independent design of catalyst molecules and reaction fields remains fundamentally challenging, and a broadly applicable host platform enabling unified photocatalyst immobilization remains to be established.

To overcome these limitations and enable broadly applicable heterogenization of molecularly defined photocatalysts without catalyst‐specific redesign, size‐exclusion‐based encapsulation within well‐defined cavities is essential. Achieving such size‐selective encapsulation requires host frameworks that combine high structural precision with mild formation conditions that allow guest molecules to be encapsulated during framework construction. Porous organic salts (POSs) formed through charge‐assisted hydrogen bonding between aromatic sulfonic acids and amines uniquely satisfy these requirements [27, 28, 29, 30]. In particular, the sodalite‐type POSs (*s*‐POSs) previously developed by our group possess three‐dimensional cage architectures with molecular‐size‐selective apertures and can encapsulate guest molecules during recrystallization, enabling size‐exclusion‐based immobilization under mild and nonreactive conditions [31, 32, 33].

**FIGURE 1:**
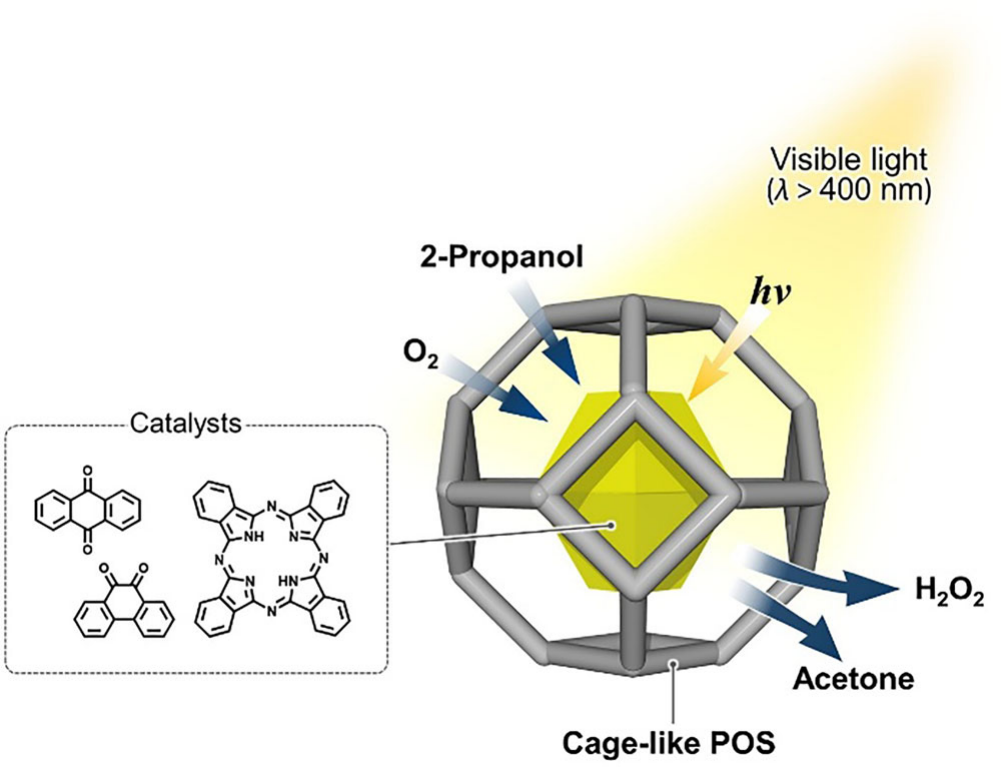
Schematic of the ship‐in‐a‐bottle strategy for photocatalyst immobilization using a cage‐type porous organic salt (POS).

In this study, we focus on an *s*‐POS constructed from adamantane tetrasulfonic acid (AdPS) and tri(ethynylphenyl)methylamine (TPMA‐^3^etn_1_) and propose a novel photocatalytic platform that leverages this material as a ship‐in‐a‐bottle‐type versatile host for molecularly defined photocatalysts, including both organic molecules and metal coordination complexes (hereafter referred to as “molecular photocatalysts”). Analogous to a ship confined within a bottle, with only light and air allowed to access the interior, the proposed system functions as a nanoreactor [34, 35, 36] that provides sufficient reaction space within the cage, while the apertures act as size‐selective barriers permitting only substrates and products to pass through (Figure 1). Moreover, a wide variety of photocatalysts with different chemical structures and properties, such as anthraquinone (AQ), phenanthrenequinone (PQ), and phthalocyanine (Pc), can be immobilized using a unified and simple recrystallization procedure under mild conditions, without the need to redesign the host framework for each catalyst. The effectiveness of this ship‐in‐a‐bottle‐type POS‐based photocatalytic platform is demonstrated through photocatalytic hydrogen peroxide production as a representative reaction [37, 38, 39, 40].

## 2 Results and Discussion

### 2.1 Construction and Structural Identification of Sodalite‐Type Cage POS

The AdPS/TPMA‐^3^etn_1_ POS platform was constructed by recrystallization, and molecular photocatalysts were incorporated into the cage interiors during analogous recrystallization processes. The resulting crystalline host and host–guest materials were characterized by X‐ray diffraction and spectroscopic analyses, followed by post‐synthetic thiol–yne crosslinking and photocatalytic evaluation of selected samples; detailed procedures are provided in the Supporting Information.

**FIGURE 2:**
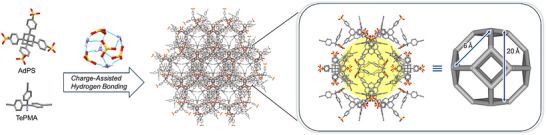
Construction of the cage‐type porous organic salt (POS) from AdPS and TPMA‐^3^etn_1_ through charge‐assisted hydrogen bonding. The resulting cage architecture is also shown. The crystalline framework features discrete cage interiors connected by molecular‐size apertures, providing confined yet accessible spaces for guest encapsulation. The highlighted cage dimensions illustrate the structural basis for size‐selective confinement within the POS framework.

The combination of AdPS and TPMA‐^3^etn_1_ afforded a cage‐type POS possessing a sodalite (*sod*) topology identical to that reported previously [31, 32, 33]. Single crystals suitable for single‐crystal XRD (SCXRD) were obtained by recrystallization of the crude salt formed from AdPS and TPMA‐^3^etn_1_. SCXRD revealed that the resulting crystal adopts a three‐dimensional *sod*‐type cage network (Figure 2). Detailed crystallographic parameters are summarized in Table S1. The crystal structure exhibits well‐defined internal cavities with a diameter of approximately 20 Å, which are interconnected through window‐like apertures of approximately 6 Å (Figure S7). These structural features indicate that the cage interior provides sufficient space to accommodate molecular photocatalysts, while the apertures serve as size‐selective openings. Based on these observations, the AdPS/TPMA‐^3^etn_1_ framework was considered a suitable host structure for cage‐confined molecular photocatalysis.

**FIGURE 3:**
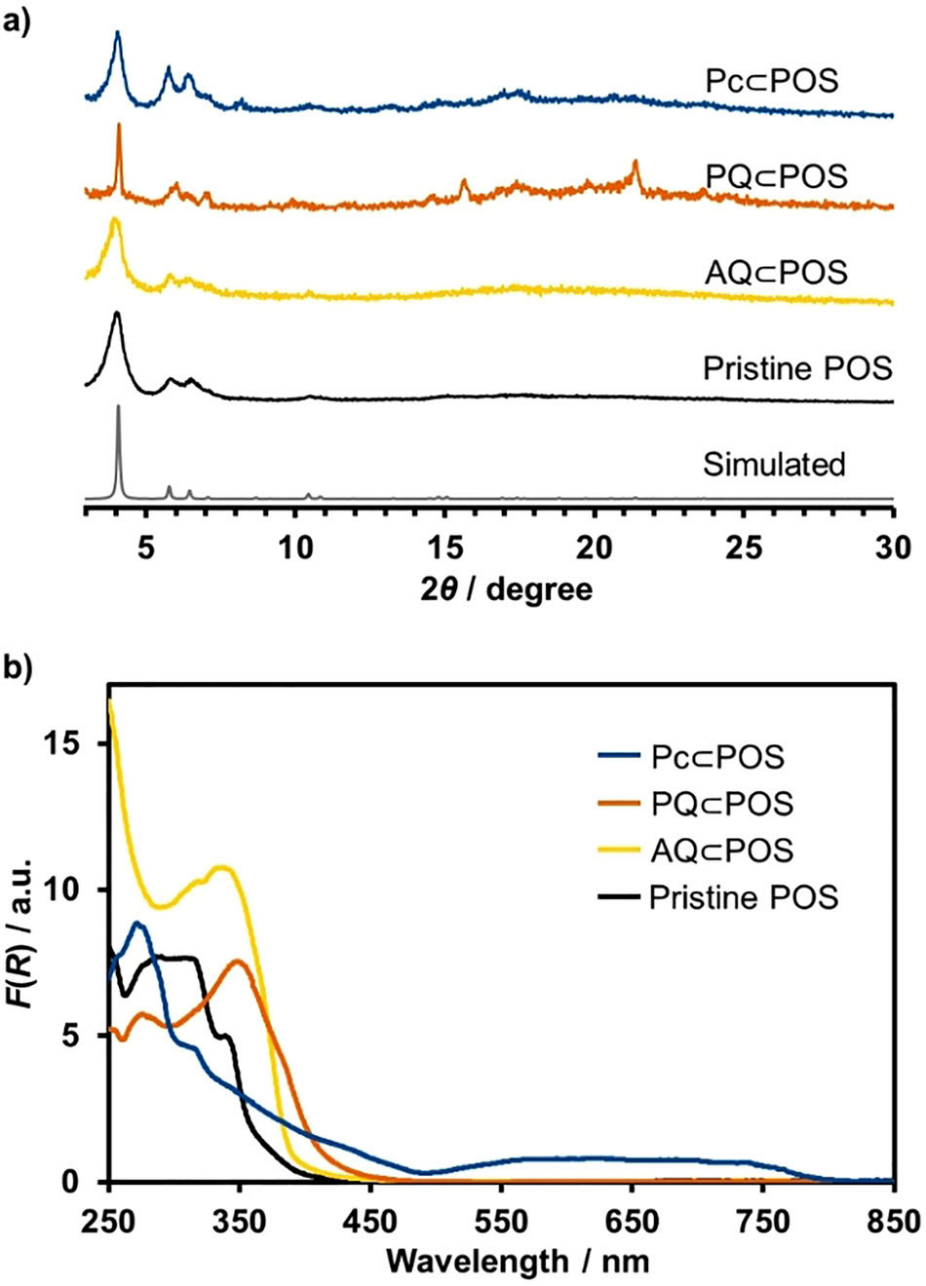
(a) Powder X‐ray diffraction (PXRD) patterns of Pristine POS and POS crystals encapsulating anthraquinone (AQ), phenanthrenequinone (PQ), and phthalocyanine (Pc). (b) Solid‐state UV–vis diffuse reflectance spectra of Pristine POS, AQ⊂POS, PQ⊂POS, and Pc⊂POS converted using the Kubelka–Munk function.

The crystallographic structure of AdPS/TPMA‐^3^etn_1_ was retained in the bulk material. Powder XRD (PXRD) patterns showed excellent agreement with the simulated pattern generated from the single‐crystal structure (Figure 3a). No additional diffraction peaks attributable to other polymorphic forms were observed. These results demonstrate that the *sod*‐type cage structure is reproducibly formed throughout the bulk material, confirming the robustness of the AdPS/TPMA‐^3^etn_1_ framework.

### 2.2 Encapsulation of Molecular Photocatalysts and Optical Properties

The AdPS/TPMA‐^3^etn_1_ framework was found to encapsulate a variety of molecular photocatalysts using a unified recrystallization protocol under mild conditions. By adding AQ, PQ, or Pc into the recrystallization solvent, crystalline samples encapsulating each photocatalyst (AQ⊂POS, PQ⊂POS, and Pc⊂POS, respectively) were obtained (Figure S8). PXRD measurements of the photocatalyst‐encapsulated samples revealed that the characteristic diffraction patterns of the sodalite‐type host structure were preserved in all cases (Figure 3a). Importantly, no significant changes in the diffraction profiles were observed despite differences in the molecular size and structure of the encapsulated photocatalysts. Because AQ, PQ, and metal‐free Pc provide limited contrast for direct spatial visualization, complementary model–guest experiments were performed using TPP and CoPc. As a complementary visualization experiment, confocal fluorescence microscopy was performed using TPP⊂POS as a fluorescent model guest‐loaded crystal (Figure S10). Fluorescence signals were observed in multiple optical sections acquired at different *z*‐positions from near the upper surface of the same crystal, as well as in the three‐dimensional reconstructed view, providing complementary visual support for the presence of TPP‐derived fluorescence within the POS crystal (Figures S11 and S12). As a complementary analysis to assess possible macroscopic surface deposition, SEM‐EDX mapping was performed using CoPc as a metal‐containing phthalocyanine model guest. Weak Co signals were detected from the crystal region, whereas no large Co‐rich surface aggregates were observed (Figure S14). These model–guest experiments support guest incorporation into POS crystals and argue against dominant macroscopic surface deposition. These results indicate that the AdPS/TPMA‐^3^etn_1_ framework can serve as a versatile host for molecular photocatalysts whose sizes are compatible with the cage dimensions.

The AdPS/TPMA‐^3^etn_1_ framework itself is transparent to visible light. The solid‐state ultraviolet–visible (UV–vis) absorption spectrum of pristine AdPS/TPMA‐^3^etn_1_ showed negligible absorption in the visible‐light region above 400 nm (Figure 3b). In contrast, AQ⊂POS, PQ⊂POS, and Pc⊂POS displayed distinct absorption bands corresponding to the encapsulated photocatalysts. No significant background absorption originating from the host framework was observed. These results indicate that visible light is efficiently transmitted through the POS framework and selectively absorbed by the encapsulated photocatalysts, enabling effective photoexcitation without competitive light absorption by the host.

The loading amount of PQ within the AdPS/TPMA‐^3^etn_1_ framework was quantitatively evaluated to assess whether the density of the encapsulated photocatalyst was sufficient to drive photocatalytic reactions while maintaining accessible free space for substrate diffusion. Based on a calibration curve for PQ (Figure S19) and UV–vis absorption measurements of dissolved PQ⊂POS samples (Figure S20), the PQ loading was estimated to be approximately 0.124 PQ molecules per cage, corresponding to 12.4 mol% relative to the number of cages.

This loading level indicates that a sufficient amount of photocatalyst is present within the cages to form an effective reaction field. Furthermore, the low occupancy suggests that substantial free volume remains inside the cages, allowing substrates and reaction intermediates to diffuse without significant steric hindrance. Thus, the encapsulation state of PQ in this system represents an appropriate balance between catalytic site density and mass transport.

### 2.3 Structural Reinforcement and Solvent Resistance by Post‐Synthetic Crosslinking

**FIGURE 4:**
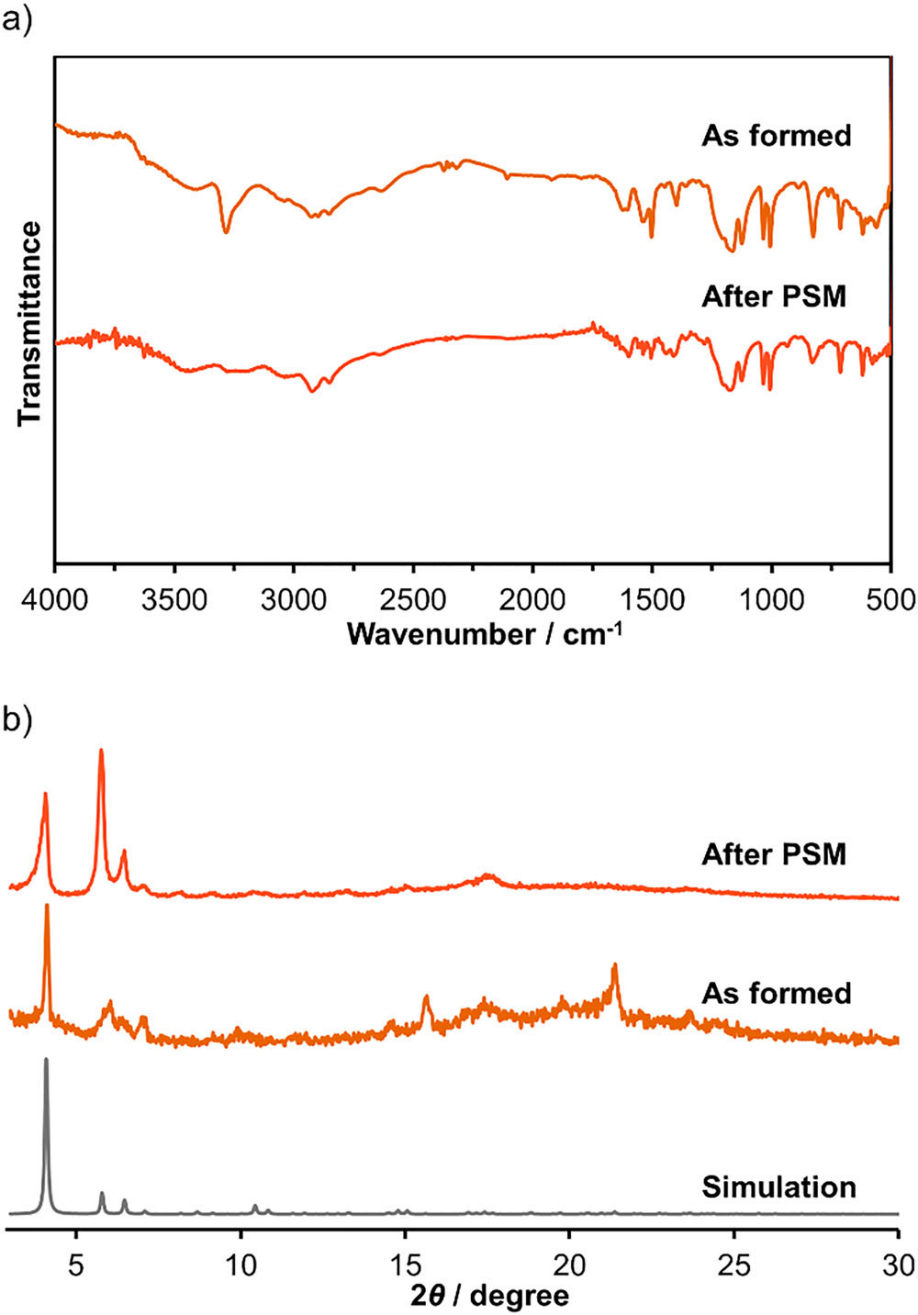
(a) IR spectra and (b) PXRD patterns of AdPS/TPMA‐^3^etn_1_ before and after post‐synthetic thiol–yne crosslinking with 1,4‐butanedithiol.

The ethynyl groups on TPMA‐^3^etn_1_ serve as reactive sites for post‐synthetic modification, enabling crosslinking after photocatalyst encapsulation. Thiol–yne crosslinking [41] was carried out using 1,4‐butanedithiol as a bifunctional linker (Figure S21). After the reaction, the characteristic IR absorption band corresponding to the ethynyl C─H stretching vibration was no longer clearly observed (Figures 4a and S22), supporting the consumption of ethynyl groups during PSM. Elemental analysis showed an increased sulfur content after PSM, which is consistent with the introduction of sulfur‐containing linkages derived from 1,4‐butanedithiol into the AdPS/TPMA‐^3^etn_1_ framework (Table S4).

The crystalline structure of AdPS/TPMA‐^3^etn_1_ was preserved after thiol–yne crosslinking. PXRD patterns of the crosslinked samples remained consistent with the simulated pattern derived from the single‐crystal structure (Figures 4b and S23), confirming structural retention. Furthermore, the crosslinked samples exhibited markedly enhanced solvent resistance and were insoluble even in highly polar solvents such as dimethyl sulfoxide (Figure S24). These results indicate that post‐synthetic crosslinking effectively reinforces the framework while maintaining structural order, thereby improving the chemical robustness of the POS host.

Although conventional N_2_ adsorption analysis was not applicable because of negligible N_2_ uptake at 77 K, apparent BET and Langmuir surface areas were estimated from CO_2_ adsorption isotherms measured at 194 K. These values should be regarded as CO_2_‐derived apparent surface areas rather than conventional N_2_‐derived surface areas. The apparent BET and Langmuir surface areas decreased after PQ encapsulation and subsequent PSM (Figures S16–S18; Tables S2 and S3), indicating that the local CO_2_‐accessible adsorption environment was modified by guest encapsulation and post‐synthetic crosslinking. Nevertheless, the CO_2_ adsorption isotherms still showed measurable uptake over the examined pressure range, and the high‐pressure CO_2_ uptake was largely retained after PSM (Figure S16). These results suggest that the ethynyl groups can serve as post‐synthetic modification sites without severely reducing the CO_2_‐accessible pore volume required for substrate diffusion and photocatalytic reactions.

### 2.4 Photocatalytic H_2_O_2_ Production and Verification of Heterogeneous Catalysis

Photocatalytic H_2_O_2_ production was examined as a proof‐of‐concept reaction to demonstrate the robustness of the cage‐based immobilization strategy. PQ⊂POS‐PSM generated hydrogen peroxide under visible‐light irradiation in the presence of isopropanol and oxygen (Figure S25). Upon visible‐light irradiation, H_2_O_2_ formation was observed, with a production rate of 4.2 mmol g^−^^1^ h^−^^1^ (Table 1). These results support that the immobilized PQ molecules contribute to the observed photocatalytic activity. Apparent TOF values estimated from the amount of PQ are summarized as reference values in Table S5.

**TABLE 1:** Control and scavenger experiments for photocatalytic H_2_O_2_ production under visible‐light irradiation. Unless otherwise noted, H_2_O_2_ production rates were normalized by the total mass of the added material.

Sample	Conditions	H_2_O_2_ production rate (mmol g^−^^1^ h^−^^1^)
No catalyst	O_2_ /visible light	0.3^a^
Pristine POS‐PSM	O_2_ /visible light	0.4
PQ⊂POS‐PSM	O_2_ /visible light	4.2
PQ⊂POS‐PSM	N_2_ /visible light	<LOD^b^
PQ⊂POS‐PSM	O_2_ /dark	<LOD^b^
PQ⊂POS‐PSM + TEMPO	O_2_ /visible light	0.2
PQ⊂POS‐PSM + BQ	O_2_ /visible light	0.8
Physical mixture^c^	O_2_ /visible light	4.6
Free PQ	O_2_ /visible light	48
Supernatant	O_2_ /visible light	0.3^a^

^a^ The value is given in mmol h^−^^1^ because no catalyst was added.

^b^ LOD = limit of detection.

^c^ The physical mixture was prepared by mixing Pristine POS‐PSM with free PQ in an amount corresponding to the PQ content in PQ⊂POS‐PSM.

H_2_O_2_ production was dependent on the presence of immobilized PQ, visible‐light irradiation, and oxygen [40]. Only a small amount of H_2_O_2_ was observed in the absence of a solid photocatalyst or when Pristine POS‐PSM was used, whereas no detectable H_2_O_2_ was observed under dark conditions or in a nitrogen atmosphere (Table 1). Scavenger experiments were further performed as complementary mechanistic probes. The addition of TEMPO or p‐benzoquinone suppressed H_2_O_2_ formation, suggesting that radical‐mediated processes are involved in the photocatalytic pathway. In addition, free PQ and physical‐mixture controls were examined under the same reaction conditions to compare PQ⊂POS‐PSM with non‐encapsulated PQ systems. These control experiments indicate that the observed H_2_O_2_ formation requires PQ and is promoted under visible‐light irradiation in the presence of oxygen. Although free PQ exhibited higher apparent photocatalytic activity, it could not be recovered and reused as a heterogeneous solid under the present conditions. The physical mixture showed a production rate comparable to that of PQ⊂POS‐PSM, indicating that encapsulation does not enhance the intrinsic molecular activity of PQ in this system. Rather, the principal advantage of PQ⊂POS‐PSM is the heterogenization of the molecular photocatalyst, enabling repeated use without detectable PQ leaching while retaining the crystalline framework.

**FIGURE 5:**
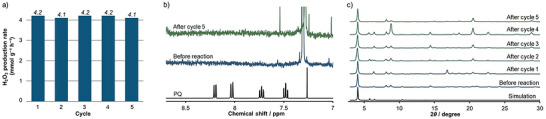
(a) H_2_O_2_ production rates of PQ⊂POS‐PSM over five cycles. (b) ^1^H NMR spectra of the supernatant solution after photocatalytic reactions using PQ⊂POS‐PSM, compared with that of free PQ. (c) PXRD patterns of PQ⊂POS‐PSM before and after photocatalytic reactions.

PQ⊂POS‐PSM functioned as a heterogeneous photocatalyst without detectable catalyst leaching. When the solid catalyst was removed from the reaction mixture during the reaction, no further H_2_O_2_ formation was observed in the supernatant solution (Table 1). In addition, recycling experiments showed that the photocatalytic activity was retained over at least five consecutive cycles without significant loss of performance (Figure 5a). ^1^H NMR analysis of the supernatant revealed no detectable signals corresponding to PQ (Figure 5b), supporting the absence of catalyst leaching. These results collectively support the heterogeneous nature and reusability of the PQ⊂POS‐PSM photocatalyst. The structural integrity of the AdPS/TPMA‐3etn1 framework was maintained after photocatalytic reactions. PXRD patterns of the recovered PQ⊂POS‐PSM samples after photocatalytic H_2_O_2_ production were identical to those obtained before the reaction (Figure 5c), indicating that the crystalline framework remained stable under the studied reaction conditions.

To further evaluate the chemical stability of sulfur species in the crosslinked framework, XPS measurements were performed before and after the photocatalytic reaction (Figure S26). The S 2p spectra exhibited similar overall profiles after the reaction, without the appearance of a distinct new high‐binding‐energy component attributable to further oxidized sulfur species. These results support that no significant sulfur oxidation occurred under the photocatalytic conditions.

## 3 Conclusion

We demonstrated that a cage‐type POS constructed from AdPS and TPMA‐^3^etn_1_ functions as a versatile host platform for homogeneous, molecularly defined photocatalysts. The *sod*‐type POS enables the encapsulation of structurally and photophysically distinct molecular photocatalysts, including AQ, PQ, and Pc, using a unified recrystallization protocol. Owing to the negligible absorption of the host framework in the visible‐light region, selective excitation of the encapsulated photocatalysts can be achieved without competitive light absorption by the host.

Furthermore, post‐synthetic thiol–yne crosslinking of ethynyl groups on the pore surface enables structural reinforcement and insolubilization of the framework after catalyst encapsulation, while preserving the cage architecture and accessible internal space. As a representative example, a PQ‐encapsulated POS exhibits reusable photocatalytic activity for visible‐light‐driven hydrogen peroxide production, with no detectable catalyst leaching and excellent structural stability.

These results establish cage‐type POSs as light‐transparent nanoreactors that provide confined yet accessible reaction environments for discrete molecular photocatalysts. Notably, in this study, the platform was demonstrated in a proof‐of‐concept manner using structurally simple phenanthrenequinone as a model catalyst. Replacing the model catalyst with more active, pre‐designed photocatalysts is expected to enhance catalytic performance. In addition, programming of the cage microenvironment through structural and post‐synthetic functionalization offers opportunities to modulate substrate selectivity and catalyst–host interactions.

## Author Contributions

**Kazuki Shiga**: conceptualization, methodology, investigation, validation, software, data curation. **Ryosuke Nishikubo**: methodology, formal analysis, investigation, resources, writing – review and editing. **Masafumi Minoshima**: methodology, formal analysis, investigation, resources, writing – review and editing. **Akinori Saeki**: methodology, resources, writing – review and editing, supervision. **Kazuya Kikuchi**: methodology, resources, writing – review and editing, supervision. **Norimitsu Tohnai**: project administration, supervision, funding acquisition, conceptualization, methodology, data curation, investigation, validation, formal analysis, resources, writing – original draft, writing – review and editing.

## Conflicts of Interest

The authors declare no conflicts of interest.

## Supporting information



The authors have cited additional references within the Supporting Information [42‐46].
**Supporting File**: anie73138‐sup‐0001‐SuppMat.docx.

## Data Availability

Crystallographic data for this study have been deposited at the Cambridge Crystallographic Data Centre (CCDC) under deposition number 2525831. The data that support the findings of this study are available from the corresponding author upon reasonable request.
