# Supplementary material for: Cage‐Type Porous Organic Salts as Ship‐in‐a‐Bottle Nanoreactors for Light‐Transparent and Reusable Molecular Photocatalysts
Source: Angew Chem Int Ed Engl. 2026 Jun 10;65(33):e6654151. doi: 10.1002/anie.6654151 (PMC13452500; doi:10.1002/anie.6654151)
Supplement: Supplementary file 1 [file ANIE-65-e6654151-s001.docx]

**Supporting Information**

Cage-Type Porous Organic Salts as Ship-in-a-Bottle Nanoreactors for Light-Transparent and Reusable Molecular Photocatalysts

K. Shiga, Dr. R. Nishikubo, Dr. M. Minoshima, Prof. Dr. A. Saeki, Prof. Dr. K. Kikuchi, Prof. Dr. N. Tohnai*

Department of Applied Chemistry, Graduate School of Engineering, The University of Osaka 2-1 Yamadaoka, Suita, Osaka 565-0871, Japan.

Email: tohnai@chem.eng.osaka-u.ac.jp (Norimitsu Tohnai)

**Table of Contents**

1. General Information
2. Materials and Instrumentation
3. Synthesis of AdPS and TPMA-^3^etn_1_
4. Preparation of Sodalite-Type Porous Organic Salts (*s*-POS)
5. Encapsulation of Molecular Photocatalysts
6. Gas Adsorption Measurements
7. Determination of PQ Loading
8. Post-Synthetic Modification by Thiol–Yne Crosslinking
9. Photocatalytic Hydrogen Peroxide Production
10. Supplementary Tables
11. Crystallographic Data
12. References

**1. General Information**

All reagents and solvents were obtained from commercial suppliers and used as received unless otherwise noted. All reactions were carried out in an ambient atmosphere unless otherwise specified.

**2. Materials and Instrumentation**

**Materials.**

Anthraquinone (AQ), phenanthrenequinone (PQ), and phthalocyanine (Pc) were purchased from Tokyo Chemical Industry Co., Ltd. and FUJIFILM Wako Pure Chemical Corporation. Methanol, dimethyl sulfoxide (DMSO), benzonitrile, N,N-dimethylacetamide (DMA), isopropanol, and other solvents were obtained from commercial suppliers and used as received unless otherwise noted.

**Instrumentation.**

Single-crystal X-ray diffraction (SCXRD) data were collected using a Rigaku XtaLAB PRO diffractometer (Rigaku Corporation, Tokyo, Japan) equipped with Cu Kα radiation (λ = 1.54187 Å).

Powder X-ray diffraction (PXRD) patterns were recorded using a Rigaku Ultima IV diffractometer (Rigaku Corporation, Tokyo, Japan).

¹H NMR spectra were recorded on an ECZ400S spectrometer (JEOL Ltd., Tokyo, Japan).

Solid-state ^13^C CP/MAS NMR spectra were recorded on a Bruker AVANCE III 600WB solid-state NMR spectrometer (Bruker Corporation, Billerica, MA, USA) operating at 600 MHz for ^1^H and 150 MHz for ^13^C under magic-angle spinning at 14 kHz.

Photoluminescence and excitation spectra were recorded on a JASCO FP-8500 spectrofluorometer (JASCO Corporation, Tokyo, Japan).

UV–vis absorption spectra were measured using a V-770 spectrophotometer (JASCO Corporation, Tokyo, Japan).

Infrared (IR) spectra were recorded on an FT/IR-4200 spectrometer (JASCO Corporation, Tokyo, Japan).

Elemental analyses for C, H, and N were performed using a Micro Corder JM10 organic micro elemental analyzer (J-SCIENCE LAB Co., Ltd., Kyoto, Japan).

Sulfur analyses were performed using an organic elemental analysis system (Mitsubishi Chemical Analytech Co., Ltd., Kanagawa, Japan).

Field-emission scanning electron microscopy (FE-SEM) and SEM-EDX measurements were performed using a JEOL JSM-IT700HR field-emission scanning electron microscope equipped with a JEOL JED-2300 energy-dispersive X-ray spectrometer. The accelerating voltage was 10 kV.

Gas adsorption measurements were performed using a BELSORP-max instrument (MicrotracBEL Corp., Osaka, Japan).

Visible-light irradiation experiments were conducted using a Kessil A160WE Tuna Blue LED lamp (Kessil, Richmond, CA, USA).

X-ray photoelectron spectroscopy (XPS) measurements were performed using an ULVAC-PHI ESCA 3057 X-ray photoelectron spectrometer (ULVAC-PHI, Inc., Chigasaki, Japan).

Fluorescence imaging experiments were performed using an Andor BC43 confocal laser-scanning microscope (Oxford Instruments, Belfast, UK) equipped with a 20 × objective lens.

**3. Synthesis of AdPS and TPMA-^3^etn_1_**

**General.**

The key organic building blocks used in this study, including the cage-forming sulfonic acid (AdPS) and amine linker (TPMA-^3^etn_1_), were synthesized according to the procedures outlined below (**Schemes S1–S6**). All reactions were carried out under the indicated conditions, and isolated products were obtained by conventional workup and purification techniques.

The identities of the synthesized compounds were primarily confirmed by NMR spectroscopy. For 1,3,5,7-tetrakis(4-phenyl)adamantane (AdPh), NMR characterization was not performed owing to its extremely low solubility in common deuterated solvents; instead, its formation was confirmed by its isolated yield, physical appearance, and successful conversion to the corresponding sulfonyl chloride and sulfonic acid derivatives in subsequent reactions.

**Tris(4-((trimethylsilyl)ethynyl)phenyl)methanol (TePMOH-TMS).**

**Scheme S1.** Synthesis of TPMOH-^3^etn-TMS_1_.

A flame-dried 200 mL three-necked round-bottom flask equipped with a thermometer, magnetic stirring bar, and dropping funnel was charged with 4-(trimethylsilyl)phenylacetylene (4-BPA–TMS, 2.18 g, 8.47 mmol) and anhydrous tetrahydrofuran (THF, 30 mL) in a nitrogen atmosphere. The reaction mixture was cooled to −90 °C, and a solution of n-butyllithium in hexane (1.6 M, 5.0 mL, 8.04 mmol) was added dropwise. The mixture was stirred at −90 °C for 1 h to generate the corresponding lithium acetylide. Next, a solution of diethyl carbonate (250 mg, 2.12 mmol) in THF (5 mL) was added dropwise at −90 °C. The reaction mixture was stirred at −90 °C for an additional 2–3 h and quenched by the addition of saturated aqueous ammonium chloride solution (35 mL). After warming to 25℃, the solvent was removed under reduced pressure, and the residue was extracted with ethyl acetate. The combined organic layers were washed with water and brine, dried over anhydrous sodium sulfate, filtered, and concentrated under reduced pressure. The crude product was purified by column chromatography on silica gel (hexane/CH₂Cl₂ = 3:2), affording TPMOH-^3^etn-TMS_1_ as a white solid (0.83 g, 1.50 mmol, 71%).

¹H NMR (400 MHz, CDCl_3_, ppm): δ = 7.39 (d, ³J_H,H_ = 8.0 Hz, 6H; Ar–H), 7.15 (d, ³J_H,H_ = 8.0 Hz, 6H; Ar–H), 2.75 (s, 1H; OH), 0.24 (s, 27H; Si–CH_3_). (**Figure S1**).

**Figure S1.** ^1^H NMR spectrum (400 MHz, CDCl_3_) of TePMOH-TMS.

**Tris(4-((trimethylsilyl)ethynyl)phenyl)amine (TPMA-^3^etn-TMS_1_).**

**Scheme S2.** Synthesis of TPMA-^3^etn-TMS_1_.

TePMOH-TMS (587 mg, 1.07 mmol) and calcium chloride (1.90 g, 17.1 mmol) were placed in a 100 mL round-bottom flask, and toluene (35 mL) was added. Aqueous hydrochloric acid (12 M, 7.13 mL, 85.5 mmol) was added dropwise, and the reaction mixture was stirred at room temperature for 5 h. After the completion of the reaction, the organic layer was separated and transferred to a 200 mL round-bottom flask containing ammonium chloride (370 mg) and concentrated aqueous ammonia (17.8 mL). The mixture was stirred overnight at room temperature. Subsequently, the reaction mixture was washed with water, dried over anhydrous magnesium sulfate, filtered, and concentrated under reduced pressure. The crude product was purified by column chromatography on silica gel using a gradient elution from hexane/ethyl acetate = 5:1 to ethyl acetate, affording TPMA-^3^etn-TMS_1_ as a solid (592 mg, 1.08 mmol, 71%).

¹H NMR (400 MHz, CDCl_3_, ppm): δ = 7.37 (d, ³J_H,H_ = 8.8 Hz, 6H; Ar–H), 7.14 (d, ³J_H,H_ = 8.8 Hz, 6H; Ar–H), 3.53 (s, 2H; NH_2)_, 0.24 (s, 27H; Si–CH_3_). (**Figure S2**).

**Figure S2.** ^1^H NMR spectrum (400 MHz, CDCl_3_) of TPMA-^3^etn-TMS_1_.

**Tris(4-ethynylphenyl)methylamine (TPMA-^3^etn_1_).**

**Scheme S3.** Synthesis of TPMA-^3^etn_1_.

TPMA-^3^etn_1_–TMS (0.988 g, 1.80 mmol) was placed in a 100 mL round-bottom flask, and potassium carbonate (1.12 g, 8.10 mmol, 4.5 equiv) was added. Upon methanol (18 mL) addition, the reaction mixture was stirred at room temperature for 1 h. After the completion of the reaction, methanol was removed under reduced pressure. The residue was extracted with ethyl acetate, and the organic layer was washed with water and brine, dried over anhydrous magnesium sulfate, filtered, and concentrated under reduced pressure, affording TPMA-^3^etn_1_ as a white solid (0.585 g, 1.77 mmol, 98%).

¹H NMR (400 MHz, CDCl_3_, ppm): *δ* = 7.43 (d, ³J_H,H_ = 8.8 Hz, 6H; Ar–H), 7.21 (d, ³J_H,H_ = 8.8 Hz, 6H; Ar–H), 3.07 (s, 3H; NH), 2.25 (s, 2H; C≡CH). (**Figure S3**).

**Figure S3.** ^1^H NMR spectrum (400 MHz, CDCl_3_) of TPMA-^3^etn_1_.

**1,3,5,7-Tetrakis(4-phenyl)adamantane (AdPh).**

**Scheme S4.** Synthesis of AdPh.

A dried 200 mL three-necked round-bottom flask was charged with 1-bromoadamantane (4.00 g, 18.59 mmol) and AlCl₃ (0.992 g, 7.44 mmol, 0.40 equiv) in a nitrogen atmosphere. Dry benzene (66.2 mL, 743.7 mmol) was added, and the mixture was stirred under N₂. Subsequently, *tert*-butyl bromide (9.99 mL, 81.81 mmol, 4.40 equiv) was added, and the reaction mixture was heated to reflux (75–80 °C) with continuous stirring. After cooling to room temperature, the reaction mixture was converted to a slurry by the addition of toluene and was poured slowly into ice water. Aqueous HCl (2 M, 10 mL) was added, and the resulting solid was collected by filtration. The solid was washed with CHCl₃ and water and dried under reduced pressure, affording AdPh as a white powder (7.36 g, 16.70 mmol, 90%).

**1,3,5,7-Tetrakis(4-(chlorosulfonyl)phenyl)adamantane (AdPSCl).**

**Scheme S5.** Synthesis of AdPSCl.

A 100 mL round-bottom flask was charged with 1,3,5,7-tetrakis(4-phenyl)adamantane (AdPh) (2.00 g, 4.54 mmol) and dichloromethane (CH₂Cl₂, 30.5 mL) at room temperature, resulting in a white slurry. Chlorosulfonic acid (HSO₃Cl) (3.02 mL, 45.4 mmol, 10 equiv) was added dropwise over 5 min. The reaction mixture was stirred at 35 °C for 75 min and then cooled to room temperature. The mixture was centrifuged for 30 min, and a yellow supernatant was carefully removed. The remaining phase was concentrated under reduced pressure to yield a yellow oil. Water was added to the residue, and the mixture was sonicated, followed by filtration to collect the solid. The solid was washed with water (2 × 2 mL) and dried under reduced pressure, affording AdPSCl as a solid (cream to white). Further washing with acetone (40 mL) afforded AdPSCl as a white powder (1.32 g, 1.58 mmol, 35%).

¹H NMR (400 MHz, CD_3_OD, ppm): *δ* = 8.09 (d, ³J_H,H_ = 8.4 Hz, 8H; Ar–H), 7.97 (d, ³J_H,H_ = 8.4 Hz, 8H; Ar–H), 2.35 (s, 12H; adamantane–CH). (**Figure S4**).

**Figure S4.** ^1^H NMR spectrum (400 MHz, CD_3_OD) of AdPSCl.

**1,3,5,7-Tetrakis(4-sulfophenyl)adamantane (AdPS).**

**Scheme S6.** Synthesis of AdPS.

1,3,5,7-Tetrakis(4-(chlorosulfonyl)phenyl)adamantane (AdPSCl) (500 mg, 0.60 mmol) was placed in a 100 mL round-bottom flask, and water (50 mL) was added. The resulting heterogeneous mixture (white slurry) was heated at reflux for 2 days. After cooling to room temperature, the solvent was removed under reduced pressure, and the residue was dried under reduced pressure, affording AdPS as a white solid (458 mg, 0.60 mmol, ~100%).

¹H NMR (400 MHz, DMSO-d*_6_*, ppm): *δ* = 7.51–7.56 (m, 16H; Ar–H), 2.07 (s, 12H; adamantane–CH). (**Figure S5**).

**Figure S5.** ^1^H NMR spectrum (400 MHz, DMSO-*d*_6_) of AdPS.

**4. Preparation of Sodalite-Type Porous Organic Salts (*s*-POS)**

AdPS and TPMA-^3^etn_1_ were dissolved in methanol at a molar ratio of 1:4.4 and stirred at room temperature. After stirring, the solvent was removed under reduced pressure, and the resulting solid was washed with diethyl ether to afford the corresponding organic salt. Formation of the organic salt was confirmed by ¹H NMR spectroscopy (**Figure S6**).

**Figure S6**. ^1^H NMR spectrum (400 MHz, DMSO-*d*_6_) of AdPS/TPMA-^3^etn_1_.

The obtained organic salt was dissolved in a mixed solvent of DMA and benzonitrile, followed by recrystallization to yield a sodalite-type cage-shaped porous organic salt (*s*-POS). By adjusting the recrystallization conditions, either single crystals suitable for SCXRD or bulk crystalline samples for various measurements were obtained (**Table S1**). The detailed pore geometry and cage dimensions of AdPS/TPMA-^3^etn_1_ are shown in **Figure S7**. Photographs and optical microscopy images of pristine and photocatalyst-encapsulated crystals are provided in **Figure S8**.

**Figure S7**. Pore geometry and size of AdPS/TPMA-^3^etn_1_.

**Figure S8**. Photographs and optical microscopy images of pristine and guest-loaded POS crystals. (a–f) Photographs and (g–l) optical microscopy images of (a,g) Pristine POS, (b,h) AQ⊂POS, (c,i) PQ⊂POS, (d,j) Pc⊂POS, (e,k) CoPc⊂POS, and (f,l) TPP⊂POS.

**5. Encapsulation of Molecular Photocatalysts**

Encapsulation of molecular photocatalysts was carried out during the recrystallization process. Benzonitrile, used as a poor solvent for recrystallization, was pre-saturated with anthraquinone (AQ), PQ, or phthalocyanine (Pc). Recrystallization under these conditions afforded the corresponding photocatalyst-encapsulated samples (AQ⊂POS, PQ⊂POS, or Pc⊂POS). The obtained crystals were washed with benzonitrile and subsequently treated with supercritical carbon dioxide to remove nonencapsulated photocatalyst molecules.

Tetraphenylporphyrin (TPP) was additionally used as a fluorescent model guest for confocal fluorescence microscopy because its excitation and emission properties were compatible with the available confocal microscopy setup. TPP-loaded POS was prepared using the same recrystallization-based encapsulation procedure and was used to visualize the spatial distribution of a fluorescent guest within the crystal. The PXRD pattern of TPP⊂POS was consistent with that of Pristine POS and with the simulated pattern of the sod-type POS framework, supporting retention of the cage-type crystalline framework after TPP loading (**Figure S9a**). Solid-state UV–vis diffuse reflectance spectra, photographs, and optical microscopy images of TPP⊂POS are shown in **Figure S9b**.

**Figure S9**. Characterization of TPP-loaded POS crystals. (a) PXRD patterns of TPP⊂POS, Pristine POS, and the simulated pattern calculated from the single-crystal structure of the sod-type POS framework. (b) Solid-state UV–vis diffuse reflectance spectra of Pristine POS and TPP⊂POS converted using the Kubelka–Munk function, together with photographs and optical microscopy images of the corresponding crystals.

Solid-state photoluminescence measurements were performed to evaluate whether TPP⊂POS could be used as a fluorescent model sample for confocal microscopy. Under the present measurement conditions, solid TPP showed almost no detectable emission, whereas TPP⊂POS displayed clear emission bands upon excitation at 420 nm (**Figure S10**). The excitation spectrum monitored at 650 nm showed features consistent with TPP-derived optical transitions. These results confirmed that TPP⊂POS provides detectable fluorescence signals suitable for confocal fluorescence microscopy.

**Figure S10**. Photoluminescence properties of TPP⊂POS. (a) Emission spectra of TPP⊂POS, solid TPP, and TPP in toluene recorded with an excitation wavelength of λ_ex_ = 420 nm. (b) Excitation spectra of TPP⊂POS and TPP in toluene monitored at an emission wavelength of λ_em_ = 650 nm.

Confocal fluorescence microscopy was then performed for TPP⊂POS. Z-stack images were acquired at different z-positions of the same crystal, and fluorescence signals were observed across the crystal region in multiple optical sections (**Figure S11** and **S12)**. Importantly, the fluorescence was not observed as a strongly surface-localized bright rim or isolated surface deposits, but rather appeared throughout the observed crystal region. Although this experiment does not provide quantitative guest distribution, the z-stack images provide complementary visual support for the presence of TPP-derived fluorescence within the POS crystal and argue against dominant macroscopic surface deposition.

**Figure S11**. Confocal fluorescence microscopy images of the same TPP⊂AdPS/TPMA-^3^etn_1_ crystal acquired at different z-positions. The z = 0 μm plane was defined near the upper surface of the crystal, and the columns correspond to representative optical sections recorded at z = 0, 10, 20, 30, and 40 μm. Rows show (a) differential phase contrast images, (b) fluorescence-channel images, and (c) merged images. Fluorescence signals were observed in multiple optical sections of the same crystal. The excitation wavelength was 638 nm.

**Figure S12**. Three-dimensional reconstructed confocal fluorescence microscopy images of the same TPP⊂AdPS/TPMA-^3^etn_1_ crystal. (a) Bright-field reconstructed view, (b) fluorescence reconstructed view, and (c) merged reconstructed view. Fluorescence signals were observed in the reconstructed crystal volume.

For SEM-EDX analysis, cobalt phthalocyanine (CoPc) was used as a metal-containing phthalocyanine model guest because the elemental contrast between the POS framework and the organic guests AQ, PQ, and Pc is limited. The Co center in CoPc serves as an elemental marker for guest-derived species, enabling assessment of possible macroscopic surface deposition. CoPc⊂POS was prepared using the same recrystallization-based encapsulation procedure. The PXRD pattern of CoPc⊂POS was consistent with that of Pristine POS and with the simulated pattern of the sod-type POS framework, supporting retention of the cage-type crystalline framework after CoPc loading (**Figure S13a**). Solid-state UV–vis diffuse reflectance spectroscopy also showed broad CoPc-derived absorption features for CoPc⊂POS (**Figure S13b**).

**Figure S13**. Characterization of CoPc-loaded POS crystals. (a) PXRD patterns of CoPc⊂POS, Pristine POS, and the simulated pattern calculated from the single-crystal structure of the sod-type POS framework. (b) Solid-state UV–vis diffuse reflectance spectra of Pristine POS and CoPc⊂POS converted using the Kubelka–Munk function, together with photographs and optical microscopy images of the corresponding crystals.

SEM and SEM-EDX measurements were then performed to examine the crystal surface and elemental distribution of CoPc⊂POS. SEM images showed crystalline surfaces without large guest-derived particles or aggregates on the external crystal surface (**Figure S14a**). Elemental mapping detected C and O signals over the analyzed crystal region, as expected for the organic POS framework (**Figure S14b,c**). Weak Co signals were also detected from the crystal region, whereas no large Co-rich domains were observed on the external surface (**Figure S14d**). The corresponding EDX spectrum showed Co-related signals together with signals from framework-derived elements (**Figure S14e**). These SEM-EDX observations provide complementary support for the presence of CoPc-derived species in the crystal region and argue against dominant macroscopic surface deposition of CoPc.

**Figure S14.** SEM-EDX analysis of CoPc⊂AdPS/TPMA-3etn1. (a) SEM image, (b) C elemental map, (c) O elemental map, (d) Co elemental map, and (e) EDX spectrum of the analyzed crystal region.

Solid-state ^13^C CP/MAS NMR spectroscopy was performed to evaluate the incorporation of PQ into the POS by examining whether the solid-state environment of PQ differs from that of neat solid PQ. Neat solid PQ showed two carbonyl carbon resonances at 179.8 and 175.1 ppm (**Figure S15**). In contrast, in PQ⊂AdPS/TPMA-^3^etn_1_, signals attributable to the carbonyl carbons of PQ were observed with peak shapes and chemical-shift positions different from those of neat solid PQ. This spectral difference is not inconsistent with the possibility that PQ molecules are not present as neat solid PQ on the crystal surface but are placed in a different local environment, possibly as a more dispersed guest state within the POS. In contrast, the 120–150 ppm region was dominated by framework-derived aromatic carbon signals, and the PQ-derived aromatic carbon signals could not be assigned. Indeed, the PQ loading was approximately 12% relative to the number of cages, corresponding to only ca. 0.4 wt% of the sample. Therefore, the solid-state ^13^C CP/MAS NMR result was interpreted as weak complementary support for the incorporation of PQ into a local environment different from that of neat solid PQ, rather than as definitive evidence for guest localization.

**Figure S15**. Solid-state ^13^C CP/MAS NMR spectra of PQ⊂AdPS/TPMA-^3^etn_1_ and neat solid PQ. (a) Full spectra and (b) expanded view of the carbonyl region.

**6. Gas Adsorption Measurements**

Prior to gas adsorption measurements, the samples were activated via a supercritical carbon dioxide treatment. Subsequently, the samples were degassed at 80 °C under vacuum for 3 h in the measurement port of a BELSORP-max instrument (MicrotracBEL). CO₂ adsorption isotherms were measured at 194 K up to a relative pressure (*P*/*P*_₀_) of 1.0 using a volumetric method (**Figure S16**). The temperature of 194 K was maintained using a dry ice–isopropanol cooling bath.

Because the present POS samples showed negligible N₂ uptake at 77 K, conventional N₂-BET and N₂-Langmuir analyses were not applicable. Therefore, CO₂ adsorption isotherms measured at 194 K were used to estimate apparent BET and Langmuir surface areas as reference values (**Figure S17** and **S18, Table S2 and S3**). These values should be regarded as CO₂-derived apparent surface areas rather than conventional N₂-derived surface areas. The apparent BET and Langmuir surface areas decreased in the order Pristine POS > PQ⊂POS > PQ⊂POS-PSM, whereas the overall CO₂ uptake capacities were largely retained after guest encapsulation and post-synthetic modification (**Figure S16**). This trend suggests that PQ encapsulation and PSM modify the low-pressure adsorption behavior and local accessible adsorption environments without completely blocking CO₂-accessible microporosity. The BET and Langmuir plots were fitted over their respective linear regions.

**Figure S16**. CO₂ adsorption isotherms measured at 194 K for (a) Pristine POS, (b) PQ⊂POS, and (c) PQ⊂POS-PSM.

**Figure S17.** Apparent BET plots derived from CO₂ adsorption isotherms measured at 194 K for (a) Pristine POS, (b) PQ⊂POS, and (c) PQ⊂POS-PSM.

**Figure S18.** Apparent Langmuir plots derived from CO₂ adsorption isotherms measured at 194 K for (a) Pristine POS, (b) PQ⊂POS, and (c) PQ⊂POS-PSM. The surface areas are reported as apparent Langmuir surface areas.

**7. Determination of PQ Loading**

The loading amount of PQ in PQ⊂POS was determined via UV–vis absorption spectroscopy. PQ⊂POS (5.0 mg) was completely dissolved in methanol (3.0 mL), and the absorbance of the resulting solution was measured. The PQ concentration was determined from a calibration curve (**Figure S19**). From the measured absorbance (A = 0.0475), the PQ concentration was calculated to be 3.3 × 10⁻⁵ M, corresponding to 9.89 × 10⁻⁸ mol of PQ in 3.0 mL.

The number of cages contained in the weighed PQ⊂POS sample was estimated from the formula weight of the cage framework. One cage consists of three fundamental ionic units composed of one sulfonic acid and four amine molecules (i.e., 3 × [acid + 4 amines]), corresponding to a total of three sulfonic acids and twelve amines per cage. The formula weight of one [acid + 4 amines] unit is 2087 g mol⁻¹; therefore, the formula weight of one cage is 3 × 2087 = 6261 g mol⁻¹. Accordingly, 5.0 mg of PQ⊂POS corresponds to 7.98 × 10⁻⁷ mol of cages.

The PQ loading was calculated as the molar ratio of PQ to cages.

$$Loading=\frac{n(PQ)}{n(cage)}=\frac{9.89\times{10}^{-8}}{7.98\times{10}^{-7}}=0.124,$$

This value corresponds to 0.124 PQ molecules per cage, or 12.4 mol% relative to the number of cages.

The calibration curve used for PQ quantification is shown in **Figure S19**, and representative UV–vis spectra are provided in **Figure S20**.

**Figure S19**. (a) Visible-light absorption spectrum of PQ in methanol. (b) Calibration curve obtained from the absorbance of PQ in methanol at 413 nm.

**Figure S20**. UV–vis absorption spectra of methanol solutions of the pristine POS, PQ, and PQ⊂POS.

**8. Post-Synthetic Modification by Thiol–Yne Crosslinking**

Post-synthetic modification (PSM) was performed via thiol–yne crosslinking using the ethynyl groups of TPMA-^3^etn_1_ as reactive sites. AdPS/TPMA-^3^etn_1_-based crystals (20 mg) were dispersed in toluene (2.0 mL), and 1,4-butanedithiol (2.0 equivalents relative to the total amount of ethynyl groups) and azobisisobutyronitrile (AIBN; 0.10 equivalents relative to the total amount of ethynyl groups) were added. The reaction mixture was heated at 80 °C for 12 h using a metal block bath while shaking at 60 rpm under air without degassing. After the reaction, the solid was collected, washed with toluene, and subjected to supercritical CO₂ treatment to remove residual reagents and solvent. A schematic illustration of the thiol–yne PSM is shown in **Figure S21**.

**Figure S21**. Schematic of post-synthetic thiol–yne modification (PSM) of the AdPS/TPMA-^3^etn_1_ framework. Ethynyl groups in the framework serve as reactive sites for reaction with 1,4-butanedithiol (BuDT), resulting in a reinforced and insolubilized framework.

The progress of PSM and the retention of the crystalline framework were first evaluated by IR spectroscopy and PXRD. After PSM, the characteristic IR band assigned to the ethynyl C–H stretching vibration was no longer clearly observed, supporting the progress of thiol–yne PSM in the AdPS/TPMA-^3^etn_1_ framework (**Figure S22**). PXRD patterns before and after PSM showed that the characteristic diffraction peaks of the sod-type POS framework were retained, indicating that the crystalline cage framework was preserved after thiol–yne modification (**Figure S23**). The PSM-treated crystals also exhibited enhanced solvent resistance after immersion in water and DMSO, supporting the reinforcement and insolubilization of the framework (**Figure S24**). Furthermore, CO₂ adsorption measurements showed that measurable CO₂ uptake was retained after PSM, indicating that CO₂-accessible microporosity was not completely blocked (**Figure S16**).

**Figure S22**. IR spectra of (a) pristine AdPS/TPMA-^3^etn_1_, (b) AQ⊂AdPS/TPMA-^3^etn_1_, and (c) Pc⊂AdPS/TPMA-^3^etn_1_ before and after PSM. Regions highlighted in blue correspond to the C–H stretching vibration of ethynyl groups in the AdPS/TPMA-3etn1 framework.

**Figure S23.** PXRD patterns of (a) pristine AdPS/TPMA-^3^etn_1_, (b) AQ⊂AdPS/TPMA-^3^etn_1_, and (c) Pc⊂AdPS/TPMA-^3^etn_1_ before and after PSM.

**Figure S24**. Optical microscopy images of AdPS/TPMA-^3^etn_1_ after PSM and subsequent solvent immersion treatment in (a) water and (b) DMSO for 10 days with sonication.

Elemental analysis was performed to assess the compositional change associated with thiol–yne PSM (**Table S4**). The experimentally determined sulfur content increased from 5.6 wt% for PQ⊂AdPS/TPMA-3etn1 to 10 wt% for PQ⊂AdPS/TPMA-^3^etn_1_ after PSM, and the C/S mass ratio decreased from 13 to 6.9. These changes are consistent with the introduction of sulfur-containing components derived from 1,4-butanedithiol into the AdPS/TPMA-^3^etn_1_ framework. Although these elemental analysis data do not indicate quantitative conversion of all ethynyl groups, the increase in sulfur content, together with the attenuation of the ethynyl C–H stretching band in the IR spectra, enhanced solvent resistance after PSM, retention of PXRD patterns, and preservation of measurable CO₂ uptake, supports that thiol–yne PSM proceeded to an extent sufficient to impart solvent resistance while retaining the crystalline framework.

In the FT-IR spectrum of unmodified Pc, an absorption feature was observed in the spectral region close to the ethynyl C–H stretching vibration, which may originate from Pc-derived vibrational contributions, such as the inner N–H stretching vibration of metal-free phthalocyanine. In the as-formed Pc⊂AdPS/TPMA-^3^etn_1_ sample, however, this region is considered to be dominated by the terminal ethynyl C–H stretching vibration of the TPMA-^3^etn_1_ framework because the amount of incorporated Pc is expected to be low, likely lower than that of smaller guests such as PQ. In addition, Pc-derived IR features are expected to overlap with framework-derived bands in this region. Therefore, the disappearance of this band after PSM was assigned mainly to the consumption of framework ethynyl groups, while possible minor Pc-derived contributions in this region could not be separately resolved. Accordingly, Pc-derived IR features were not used as primary evidence for either guest incorporation or quantitative evaluation of PSM.

**9. Photocatalytic Hydrogen Peroxide Production**

Photocatalytic reactions were carried out upon visible-light irradiation. In the standard photocatalytic H₂O₂ production experiment, PQ⊂POS-PSM (20 mg) was dispersed in isopropanol (5.0 mL) in a glass sample tube. The tube was connected to an O₂ balloon to maintain an oxygen atmosphere throughout the reaction. The suspension was continuously and vigorously mixed using a vortex mixer operated at its maximum setting to maintain a uniform suspension of the solid catalyst during the reaction.

Visible-light irradiation was provided by a blue LED with an emission maximum at 440–450 nm (Kessil A160WE Tuna Blue, maximum power consumption: 40 W), positioned approximately 10 cm from the reaction vessel (**Figure S25**). The light intensity at the reaction position was not independently calibrated; however, all experiments were conducted under identical geometric and instrumental conditions to ensure reproducibility. The reaction was conducted at ambient temperature (20–25 °C) without external temperature control; care was taken to minimize heat accumulation from the light source.

**Figure S25**. Photocatalytic reaction setup. (a) Photograph of the experimental setup used for visible-light-driven photocatalytic reactions. (b) Schematic of the photocatalytic reaction system.

After visible-light irradiation, hydrogen peroxide (H₂O₂) was quantified by iodometric redox titration according to a procedure reported for anthraquinone-based photocatalytic systems,^[37]^ with minor modification in the concentration of the titrant. Specifically, 2.0 mL of the reaction solution was transferred to a beaker, to which 5.0 mL of 2.0 M H₂SO₄, 4.0 mL of KI solution (100 g L⁻¹), and three drops of 5 wt% ammonium molybdate solution were added. The mixture was covered and kept in the dark for 5 min to allow the complete oxidation of iodide by H₂O₂. The liberated iodine was titrated with a standardized aqueous sodium thiosulfate solution (0.01 M) using starch solution (10 g L⁻¹) as an indicator, and the endpoint was determined by the disappearance of the blue color. The original literature procedure employed 0.10 M Na₂S₂O₃; however, in this work, a 0.01 M solution was used to improve titration resolution over the present concentration range.

The photocatalytic reaction for the first cycle was performed in triplicate (n = 3), and the average value was considered. Subsequent recycling experiments were conducted once per cycle. Recycling experiments were performed by recovering the solid catalyst by filtration, washing with isopropanol, and reusing it under identical conditions. Free PQ was examined as a non-encapsulated molecular control under the same reaction conditions. The physical mixture was prepared by mixing Pristine POS-PSM with free PQ in an amount corresponding to the PQ content in 20 mg of PQ⊂POS-PSM. Unless otherwise noted, 20 mg of the added material was used for each photocatalytic experiment.

Scavenger experiments were carried out under the same photocatalytic conditions as the standard H₂O₂ production experiment. TEMPO and p-benzoquinone were added at a concentration of 10 mM as scavengers to probe the involvement of radical-mediated processes. After visible-light irradiation under an O₂ atmosphere, the amount of H₂O₂ was quantified by iodometric titration using the same procedure as that used for the standard photocatalytic reaction.

Apparent TOF values were estimated from the amount of PQ and are summarized in **Table S5**. The H₂O₂ production rates were normalized by the total mass of the added material, whereas the apparent TOF values were calculated based on the amount of PQ. For PQ⊂POS-PSM-based samples, the PQ loading was estimated as 12% relative to the number of cages, with one cage corresponding to three AdPS units. Because the amount of PQ is small relative to the framework mass, the calculated values are sensitive to assumptions regarding the framework composition and the extent of thiol–yne post-synthetic modification. Therefore, these values are provided as reference values. For free PQ, the TOF was calculated based on the amount of added PQ. For the physical mixture, the TOF was calculated based on the amount of free PQ mixed with Pristine POS-PSM.

XPS measurements were performed to evaluate possible changes in the chemical states of sulfur species in PQ⊂AdPS/TPMA-3etn1 after PSM before and after the photocatalytic reaction (**Figure S26**). The AdPS/TPMA-^3^etn_1_ framework after PSM contains sulfur atoms originating from both the sulfonate groups of AdPS and the thioether-like linkages introduced by thiol–yne modification; therefore, the S 2p region may contain overlapping contributions from different sulfur environments. Although quantitative deconvolution of these components was not performed, the overall S 2p spectral envelope showed no significant change after the photocatalytic reaction. In particular, no distinct additional high-binding-energy component attributable to further oxidized sulfur species was observed.

**Figure S26**. XPS spectra of PQ⊂AdPS/TPMA-^3^etn_1_ after PSM before and after the photocatalytic reaction: (a) O 1s, (b) C 1s, and (c) S 2p regions.

**10. Supplementary Tables**

**Table S1**. Crystallographic parameters of AdPS/TPMA-^3^etn_1_.

| **Crystal** | **AdPS/TPMA-^3^etn_1_** |
| --- | --- |
| Crystal system | *Cubic* |
| Space group | *P*4_2_32 (#208) |
| *a* (Å) | 30.8632(3) |
| *b* (Å) | 30.8632(3) |
| *c* (Å) | 30.8632(3) |
| *α* (°) | 90 |
| *β* (°) | 90 |
| *γ* (°) | 90 |
| *V* (Å^3^) | 29398.3(5) |
| *R*1 | 0.0464 |
| *wR*2 | 0.1259 |
| GOF | 0.8317 |
| Completeness | 0.999 |
| Temperature (K) | 213 |

**Table S2.** Apparent BET surface areas of Pristine POS, PQ⊂POS, and PQ⊂POS-PSM estimated from CO₂ adsorption isotherms measured at 194 K.

| **Sample** | **Linear range, *P*/*P*₀** | ***R*²** | ***V*_m_ / cm³(STP) g⁻¹** | ***C*** | **Apparent *S*_BET_ / m² g⁻¹** |
| --- | --- | --- | --- | --- | --- |
| Pristine POS | 0.0501–0.2632 | 0.9982 | 56.0 | 36.7 | 256 |
| PQ⊂POS | 0.0638–0.2157 | 1.000 | 44.1 | 48.0 | 202 |
| PQ⊂POS-PSM | 0.0703–0.2684 | 0.9919 | 29.2 | 9.1 | 134 |

**Table S3.** Apparent Langmuir surface areas of Pristine POS, PQ⊂POS, and PQ⊂POS-PSM estimated from CO₂ adsorption isotherms measured at 194 K. K is the apparent Langmuir constant obtained using relative pressure *P*_e_/*P*_0_.

| **Sample** | **Langmuir range, *P*_e_/*P*_0_** | ***R*²** | ***V*_m_**  **/ cm³(STP) g⁻¹** | **K** | **Apparent *S*_Langmuir_**  **/ m² g⁻¹** |
| --- | --- | --- | --- | --- | --- |
| Pristine POS | 0.0501–0.2632 | 0.9845 | 87.5 | 14 | 400 |
| PQ⊂POS | 0.0638–0.2157 | 0.9958 | 64.4 | 18.6 | 294 |
| PQ⊂POS-PSM | 0.0703–0.2178 | 0.9786 | 49.9 | 4.95 | 228 |

**Table S4**. Calculated and experimentally determined elemental compositions and C/S mass ratios of PQ⊂POS before and after PSM.

| **Model/sample** | **C / wt%** | **H / wt%** | **N / wt%** | **S / wt%** | **C/S mass ratio** |
| --- | --- | --- | --- | --- | --- |
| Before PSM, model | 77 | 4.8 | 2.7 | 6.2 | 13 |
| Before PSM, found | 75 | 5.3 | 2.8 | 5.6 | 13 |
| After PSM, model | 67 | 5.7 | 2.0 | 18 | 3.7 |
| After PSM, found | 69 | 5.3 | 2.4 | 10 | 6.9 |

**Table S5**. H₂O₂ production rates and apparent TOF values for photocatalytic H₂O₂ production under different reaction conditions. The H₂O₂ production rates were normalized by the total mass of the added material, whereas the apparent TOF values were estimated from the amount of PQ.

| **Sample** | **Conditions** | **H₂O₂ production rate**  **(mmol g⁻¹ h⁻¹)** | | **TOF (h^-1^)** |
| --- | --- | --- | --- | --- |
| PQ⊂POS-PSM | O_2_ / Visible light | | 4.2 | 2.2 x 10^2^ |
| PQ⊂POS-PSM | N_2_ / Visible light | | <LOD ^a^ | - |
| PQ⊂POS-PSM | O_2_ / Dark | | <LOD ^a^ | - |
| PQ⊂POS-PSM + TEMPO | O_2_ / Visible light | | 0.2 | 10 |
| PQ⊂POS-PSM + BQ | O_2_ / Visible light | | 0.8 | 42 |
| Physical mixture | O_2_ / Visible light | | 4.6 | 2.4 x 10^2^ |
| Free PQ | O_2_ / Visible light | | 48 | 10 |

^a^ LOD = limit of detection.

**11. Crystallographic Data**

The SCXRD data for AdPS/TPMA-^3^etn_1_ were collected using a Rigaku XtaLAB PRO diffractometer with Cu Kα radiation (λ = 1.54187 Å) at 213 K. The crystal belongs to the cubic system with space group P4₂32 (No. 208), with unit-cell parameters *a* = *b* = *c* = 30.8632(3) Å and *α = β = γ* = 90°. The structure was solved by intrinsic phasing using SHELXT and refined by full-matrix least-squares on *F*² using SHELXL in Olex2.^[42–44]^ Diffuse electron density arising from highly disordered solvent molecules in the pores was treated using the SQUEEZE routine implemented in PLATON.^[45]^ All nonhydrogen atoms of the ordered framework were refined anisotropically where appropriate. Hydrogen atoms were placed in calculated positions and refined using a riding model. The final *R* indices were *R*1 = 0.0464 for reflections with *I* > 2σ(*I*) and *wR*2 = 0.1259 for all data, with a goodness-of-fit on *F*² of 0.8317. The SQUEEZE treatment was applied to unresolved solvent-accessible electron density and did not alter the assigned connectivity or sod-type topology of the AdPS/TPMA-^3^etn_1_ framework.

To address the checkCIF alerts noted during the review process, SCXRD data were newly collected and the structure was re-refined. The revised crystallographic model reduced the number of level B alerts to two. The remaining level B alerts correspond to PLAT230 Hirshfeld difference alerts for C00T–C00Z and C00X–C012. These alerts are attributed to non-ideal anisotropic displacement parameters in locally disordered regions of the framework, particularly around peripheral organic components facing the solvent-accessible pores. Such local displacement-parameter issues are reasonable for an all-organic porous salt containing large solvent-accessible voids and highly disordered pore solvent. Detailed responses to these residual alerts were provided in the validation response form. Importantly, these local alerts do not affect the assigned framework connectivity, cubic symmetry, or sod-type cage topology, which are the structural features relevant to the conclusions of this study.

Crystallographic data for AdPS/TPMA-^3^etn_1_ have been deposited with the Cambridge Crystallographic Data Centre as CCDC 2525831.^[46]^

**12. References**

[42]	O. V. Dolomanov, L. J. Bourhis, R. J. Gildea, J. A. K. Howard, H. Puschmann, “OLEX2: a complete structure solution, refinement and analysis program” *Journal of Applied Crystallography* **42**, (2009): 339–341.

[43]	G. M. Sheldrick, “SHELXT – integrated space-group and crystal-structure determination” *Acta Crystallographica Section A: Foundations and Advances* **71**, (2015): 3–8.

[44]	G. M. Sheldrick, “Crystal structure refinement with SHELXL” *Acta Crystallographica Section C: Structural Chemistry* **71**, (2015): 3–8.

[45]	A. L. Spek, “PLATON SQUEEZE: a tool for the calculation of the disordered solvent contribution to the calculated structure factors” *Acta Crystallographica Section C: Structural Chemistry* **71**, (2015): 9–18.

[46]	Deposition number 2525831 contains the supplementary crystallographic data for this paper. These data are provided free of charge by the joint Cambridge Crystallographic Data Centre and Fachinformationszentrum Karlsruhe Access Structures service.
